# Inguinal Hernia Operated on under Local Anaesthesia

**Published:** 1911-12

**Authors:** A. G. Little

**Affiliations:** Valdosta, Ga.


### INGUINAL HERNIA OPERATED ON UNDER LOCAL ANAESTHESIA.

By A. G. Little, M. D., Valdosta, Ga.

One of the principal obstacles to surgery is the patient's fear of the anaesthetic. That fear is not without foundation, as there is, with all anaesthetics, a certain per cent. of fatalities.

In order that an anaesthetic may be accepted by the profession it must embrace the followng points:

(1) Safety to the life of the patient.

(2) Must insure a minimum amount of pain for the patient.

(3) Must permit of thorough work.

Cocaine in inguinal hernia meets these three requirements. There is practically no danger to the life of the patient, as the quantity used is much less than a lethal dose. The amount of pain experienced by the patient in the greater per cent. of cases is practically nil. The degree of anaesthesia is quite sufficient to do thorough work, and also to remedy any complication that may be found.

Also, the anatomy of this region is admirably suited for cocaine anaesthesia, as the principal structures met with in the operation are: The skin, superficial fat, apponeurosis of external oblique muscle, iliohypogastric, ilio-inguinal, genitocrucal nerve, and cord with hernial sac and contents, and of all these structures but two are necessary to be cocainized, the skin and the three nerves. By injecting very superficially the skin and the three nerves, or as many as can be found, you will have produced complete anaesthesia of the entire field of operation. In most cases all three nerves cannot be found but fortunately the most important, the ilio-inguinal, is the most constant; so the cocainizing of this nerve renders the field practically painless except the cord, which, if the genitocrucal fails to make its appearance, can be done by injecting the supeıficial structure of the cord, then the only part left for injection is the muscular fibers around the internal ring. So with all these structures thoroughly cocainized, you can do as satisfactory and thorough work as you can under general anaesthesia, without any of the unpleasant and objectionable after effects.

The solution I use is made by dissolving 2½ grains of muriate of cocaine in two ounces of normal salt solution, which makes practically a one-quarter of a one per cent. solution. Of this solution I use about one-half or three-quarters of a grain, and in no case have I had any bad results that I could trace to the effects of cocaine.

The following are histories of some cases that I used cocaine as an anaesthetic:

Case I.—Mr. M. (white). Age, twenty-seven. Height, 5 feet 8 inches. Weight, 130 pounds. General health, fair. Double indirect inguinal hernia. Right of six and left of two years' standing. The right I operated on first, the left about six weeks later.

The usual preparation before the operation and during operation were gone through with.

The line of incision was cocainized with one-quarter of one per cent. solution, with same injected into the superficial fat directly under the incision. The incision through the skin and the superficial fat was made without any pain, except a slight twinge when a blood vessel, which is accompanied by a nerve filament, was cut. The apponeurosis of the external oblique was exposed and fibers separated from external ring to upper angle of incision. Two of the nerves were distinctly seen, into the sheath of which I injected a drop of the cocaine solution; I then retraced the internal oblique upward, exposing the cord and sac, the superficial structure of which I injected, also the margin of the internal ring; I then, without any pain to the patient, separated the sac from the cord, opening and reducing contents; I both tied and cut off sac at internal ring. I then began the formation of a new canal for the cord by bringing the arched fiber of the internal oblige, and the conjoined tendon under the cord and suturing them by interrupted suture to Poupart's ligament, with medium sized kangaroo tendon. Then I brought the flaps of the apponeurosis of the external oblique over the cord, suturing them with No. 1 10-day chromic cat-gut. The superficial fat and skn were brought together with interrupted suture of No. 1 10-day chromic cat-gut.

I dressed the wound by applying a coating of styptic collodion, and then the usual dressing.

In this case I had to stitch abscess, which I opened and washed out. The abscess was superficial, consequently no damage was done to deep stitches.

Patient dismissed in four weeks with abscess healed and hernia cured.

Case II.—The operation for the hernia on the left side was done about six weeks after the first. The usual preparation before and during operation was gone through with.

The operation was as follows: Line of incision, which was about three inches long and the superficial fat, were cocainized; the incision through the same was painless. Going through these structures I exposed the external oblique, the fibers of which I separated from external ring to upper angle of incision; reflecting these I brought to view the fiber of the internal oblique on the surface of which I found a nerve, which I injected. Then retracing the internal oblique I exposed the cord, sac and internal ring; cocainizing the superficial structure and fibres around the internal ring, I proceeded to separate the sac, which I found very short, from the cord. This I had no trouble in doing, as it gave the patient no pain. After separating and opening the sac, contents of which I found had been reduced, I tied and cut off sac. I then brought the fibres of the internal oblique and conjoined tendon under the cord and sutured them to Poupart's ligament with interrupted stitches of medium sized kangaroo tendon. I then brought flaps of apponeurosis of external oblique over cord, suturing them with No. 1 10-day chromic catgut. The superficial fat and skin I closed with interrupted sutures of No. 1 10-day chromic cat-gut.

In this case I got healing by first intention and dismissed patient in three weeks, cured.

Case III.—Mr. W. (white). Age 60 years. Height 5 feet 6 inches. Weight, 145 pounds. Occupation, merchant.

Right indirect inguinal hernia of about twenty years standing. Truss worn but not sufficient at all times to keep hernia up. Right hydrocele for about five years. Two months before operation hydrocele tapped, removing about one and a half pints of straw colored fluid. Afterwards twenty drops of pure carbolic acid injected into the hydrocele sac, but fluid partially returned.

Patient came to me with right hernia and right hydrocele.

Prepared field of operation in usual way.

I first tapped hydrocele, removing about one pint of fluid, and then injected about thirty drops of pure carbolic acid. The pain and shock experienced from this injection was more pronounced than I expected.

I proceeded to inject my line of incision and superficial fat, but found it necessary to give a hypodermic injection of one-quarter grain morphine to overcome shock produced by the injection of the carbolic acid. I waited until my hypodermic began to have effect and then proceeded to make incision through skin and superficial fat down to external oblique muscle. I separated fibres of external oblique from external ring to upper angle of incision. Looking up the nerves in field, I cocainized same. Retracing internal oblique upward I exposed cord, sac and contents, cocainizing the superficial structures of cord and sac, and muscular fibres around internal ring. I proceeded to separate sac from cord, which was done with very little difficulty and pain. Sac was opened, the contents, which were omentum, was reduced, and sac tied off at internal ring with No. 2 plain cat-gut, and clipped off. I then brought the fibres of the internal oblique and the conjoined tendon under the cord and sutured them to Poupart's ligament by six interrupted sutures of medium-sized kangaroo tendon. I brought together the separated fibers of the external oblique and sutured them with No. 1 ten-day chromic cat-gut, interrupted sutures. The superficial fat and the skin I brought together by a continuous stitch of No. 1 plain cat-gut.

In dressing, I first applied coating of collodion, and then the sterile gauze dressing held in place by adhesive strap and spika bandage.

In this case I had some trouble at first from the pain from the injection of carbolic acid into the hydrocele sac, but that I soon overcame by the hypodermic of morphine.

The after treatment was the same as in other cases.

Patient dismissed in three weeks. Cured.

Case IV.—Mr. C. (white). Age thirty. Height, 5 feet 10 inches. Weight, 215 pounds.

Hernia of twelve years' standing. Truss worn, but result not good.

The usual preparation of the field of operation gone through with.

Operation: Line of incision and superficial fat cocainized. No pain in going through skin and fat down to external oblique. I found that retracing sides of incision gave some pain, which was hard to overcome. I separated fibres of external oblique from external ring to upper angle of wound, retracing flaps. I found one nerve passing on outer border of internal oblique; this I cocainized, and retracing internal oblique I exposed cord with sac and contents. The cord I treated in usual way, by injecting the superficial structures and the tissue around the internal ring. I then separated sac from cord, giving very little pain; this I opened. finding contents reduced. I tied off at internal ring with No. 2 plain cat-gut and cut off. I then formed new canal for cord by bringing arched fibres of internal oblique, and the conjoined tendon under the cord and suturing them to Poupart's ligament by interrupted sutures of medium-sized kangaroo tendon; over the cord then I brought the separated fibre of the external oblique, bringing them together by means of interrupted sutures of No. 1 ten-day chromic catgut. I brought together the superficial fat and skin by buried continuous No. 1 plain cat-gut suture.

Dressing: I applied coating of collodion, then usual sterile gauze dressing.

In this case patient seemed to suffer pain when any retracing of the incision was done.

Patient dismissed in three weeks. Cured.

I have learned the following from my experience:

That one-quarter of one per cent. solution is amply sufficient to produce complete local anaesthesia.

The amount I found necessary to use in any operation produced no bad effects.

Thin people are better subjects than fat ones.

Inspire in your patient confidence in your truthfulness and ability.

Tell them when you know you are going to give them pain, as they will be expecting it then.

Arrange your patient on the table so he cannot see anything that you are doing.

Don't ask for instruments, but have them near, where you can reach them.

Keep your patient's attention diverted as much as possible by talking to him.

That neither the number, size nor position of the three principal nerves are constant.

That the lower angle of the incision is the most sensitive part of the incision, and is the most liable to give trouble from hemorrhage.

That the handling of the peritoneum, omentum or intestines gives practically no pain.

The following, I think, are some very strong points in favor of local anaesthesia:

1. Avoids a general anaesthetic, which has its dangers to life.
2. There is practically no danger to the life of patient.
3. It is more inviting to patient.
4. No nausea after operation.
5. No pain after operation.
6. Full diet in at least twelve hours after operation.

---

### SURGICAL SUGGESTIONS.

Tuberculosis of the bone develops in the epiphyses or the joint synovia. An inflammatory lesion in the shaft of a long bone is never tuberculous.

The sooner a long bone is opened in acute osteomyelitis the less the destruction.

The removal of a wedge of skin at the side of an ingrown nail, as in Cotting's operation, is rarely necessary and usually objectionable. Granulations disappear quickly when the nail segment is withdrawn; if they are exuberant they may be snipped or burned off.—American Journal of Surgery.

